# Adopting global tools for the advancement of pharmacy practice and workforce in Saudi Arabia

**DOI:** 10.1016/j.jsps.2022.05.007

**Published:** 2022-06-02

**Authors:** Dalia Almaghaslah, Asmaa Al-Haqan, Ahmed Al-jedai, Abdulrhman Alsayari

**Affiliations:** a Department of Clinical Pharmacy, College of Pharmacy, King Khalid University, Abha, Saudi Arabia; b Kuwait University, Faculty of Pharmacy, Department of Pharmacy Practice, Safat, Kuwait; c Therapeutic Affairs Deputyship, Ministry of Health, Riyadh, Saudi Arabia; d Colleges of Medicine and Pharmacy, Alfaisal University, Riyadh, Saudi Arabia; e Department of Pharmacognosy, College of Pharmacy, King Khalid University, Abha, Saudi Arabia

**Keywords:** Competency framework, Saudi Arabia, Pharmacists, International Pharmaceutical Federation (FIP), Pharmacy workforce

## Abstract

**Background:**

The continuing expansion of the pharmacist’s role necessitates continuous evaluation of current practice to identify strategies for improvements. The International Pharmaceutical Federation (FIP) has developed tools to support stakeholders in identifying development needs and planning advancement strategies. The aim of this research was to utilise the FIP Global Competency Framework, version 2 (GbCF v2), and FIP Development Goals (DGs) to evaluate competencies related to pharmacy practice in Saudi Arabia, and to understand the strategies needed to develop and improve the current practice.

**Methods:**

The study involved four phases. Phase 1 involved translation of the FIP GbCF v2 into the Arabic language. Phase 2 was a consensus panel validation to establish the initial relevance of the competencies to current practice. Phase 3 included a national survey distributed to all registered pharmacists in Saudi Arabia. The final phase was conducted through mapping ‘not relevant’ competencies to FIP DGs to identify priorities.

**Results:**

The translation phase yielded a bilingual framework that could be utilized by pharmacists in Saudi Arabia. The initial validation phase identified 61 behavioral statements (from 124 in the GbCF v2) as ‘highly relevant’ or ‘relevant’ to pharmacy practice. Findings from the national survey identified a list of ‘not relevant’ competencies that could highlight gaps in current practice. The final mapping phase generated a list of three FIP DG priorities: DG5 (competency development), DG8 (working with others) and DG11 (impact and outcomes).

**Conclusion:**

The study indicated that competencies in the GbCF v2 were relevant to pharmacists practicing in the country. However, some competencies were perceived as ‘not relevant’ to current practice and these highlighted gaps in the current practice that need attention. Mapping ‘not relevant’ competencies to FIP DGs should be used as a starting point towards developing strategies, systems, and protocols to advance pharmacy practice in Saudi Arabia.

## 1 Introduction

Ongoing emphasis is currently being placed on healthcare professionals to demonstrate specific competencies (Shah et al., 2016). With the increasing complexity of patient care, pharmacists are expected to gain and maintain knowledge, skills, and values necessary for minimizing medication related risks, as well as optimizing therapeutic outcomes (Udoh et al., 2018).

Competency is defined as the “knowledge, skills, attitudes and behaviors that an individual develops through education, training, development and experience.” These competencies can be combined into a framework that can support practitioner development within an individual for effective and sustained performance (Meštrović et al., 2012). Countries around the globe have developed or adapted competency frameworks for their pharmaceutical workforce i.e. pharmacists and pharmacy technicians (Udoh et al., 2018, Mucalo et al., 2016, Al-Haqan et al., 2020, Suwannaprom and Kessomboon, 2020, Koehler et al., 2018, Andrew et al., 2012, Meilianti et al., 2021).

**Fig. 1:**
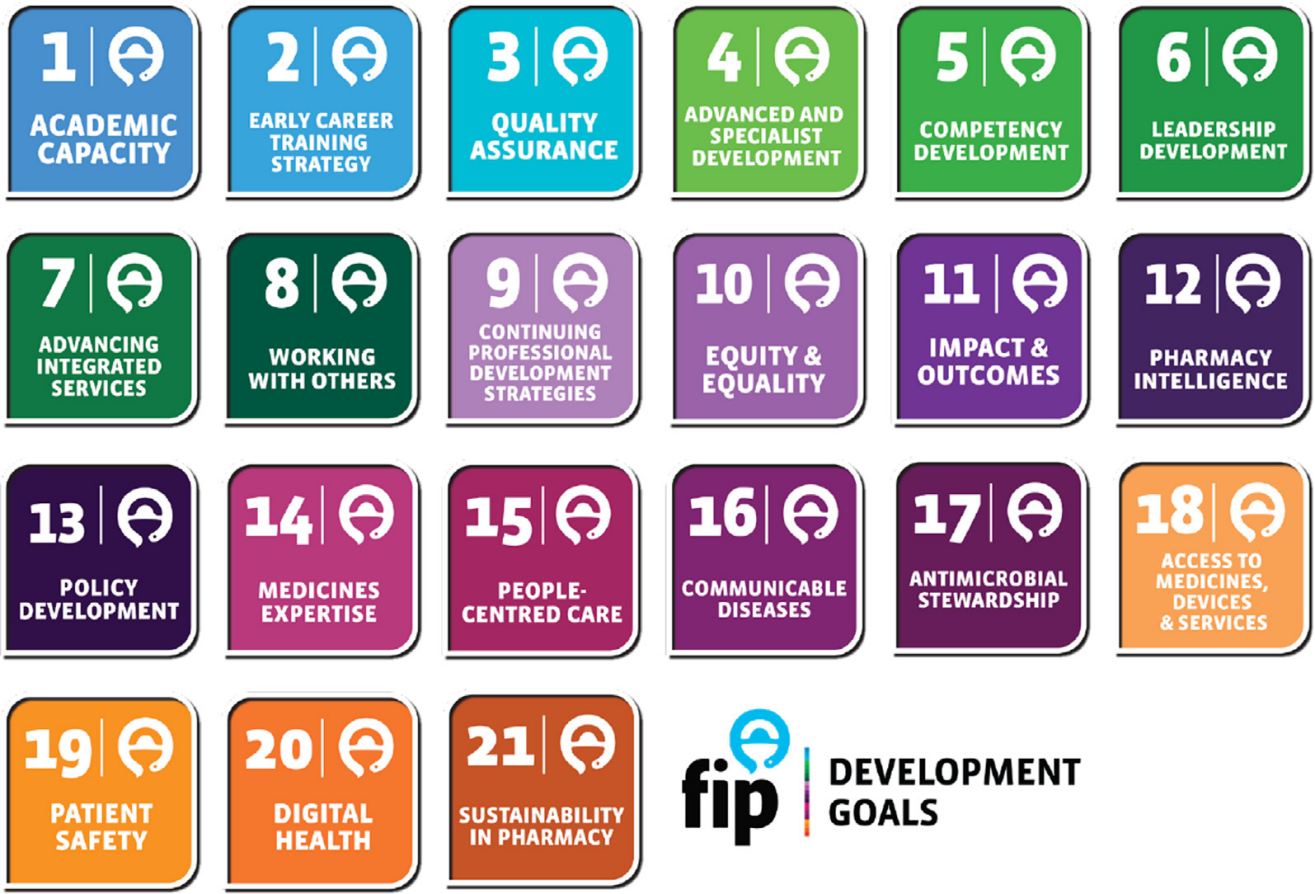
FIP development goals.

The International Pharmaceutical Federation Education Initiatives (FIP *E*d) developed an evidenced-based Global Competency Framework (GbCF v1) in 2012 containing a structured combination of behavioral competencies that can assist early career pharmacists with <5 years of practice experience (Mucalo et al., 2016). The GbCF v1 is a validated framework meant to support pharmacists to progress towards effective and sustainable performance and to contribute towards advanced practice. The framework originally comprised 100 behavioral statements integrated under 20 competency domains and four broad competency clusters; these are generally applicable for the global pharmacy workforce and adaptable to local contexts. In 2020, an updated version of the FIP GbCF, named GbCF v2, was released to support the expansion in the role of pharmacists, the wider scope of pharmaceutical services provided to patients worldwide, and the advancement of technology and therapeutics. The number of behavioral competencies increased to 124, with 23 competency domains, but remained assembled within four broad competency clusters (FIP. FIP Global Competency Framework, 2020). In the same year, the FIP published the global 21 Development Goals (DGs) as a systematic and integrated framework to guide pharmacy development and facilitate the needs-based assessment and transformation of pharmaceutical practice, science, workforce and education (International Pharmaceutical Federation, 2020). The FIP DGs (Fig. 1) were developed with the intention to provide systematic guidance for the progress and advancement of pharmacy practice and workforce globally, as well as at the country level. The FIP GbCF and DGs have been utilized in several countries to guide national practice and workforce advancement and transformation (Udoh et al., 2018, Meštrović et al., 2012, Al-Haqan et al., 2020, Meilianti et al., 2021, Al-Haqan et al., 2021, Almaghaslah and Alsayari, 2021, Meilianti et al., 2021, Udoh et al., 2021).

The pharmacy profession in Saudi Arabia has been undergoing numerous changes recently (Almaghaslah et al., 2019). The laws that regulate pharmacy practice in various settings have been updated. Over the past two decades, policy makers have focused on meeting the increasing demand for pharmaceutical services by increasing the number of the local pharmacy workforce (AlRuthia et al., 2018). The quality of pharmacy education in the country has been another area of attention. Local accreditations of the pharmacy programs by the National Centre for Academic Accreditation and Evaluation (NCAAA) have been made mandatory to assure the quality of education offered. Some pharmacy colleges went further in advancing the quality of education by obtaining international accreditation from organizations such as the Accreditation Council for Pharmacy Education (ACPE) and The Canadian Council for Accreditation of Pharmacy Programs (CCAPP) (Almaghaslah et al., 2019). Another focus of pharmacy regulatory bodies was the employment regulations. The Ministry of Human Resources and Social Development launched a labor reform initiative to re-nationalize the profession (SFDA. Saudi Code of Pharmaceutical Promotional Practices in the Kingdome of Saudi Arabia, 2012, HRSD. Renationalisation of the pharmacy profession. Published, 2020).

Currently, the continuing education requirements for pharmacists’ re-registration is lacking a systematic approach to identifying pharmacists' personal educational needs and the core competencies required for practicing in different settings. No competency frameworks exist for pharmacists in Saudi Arabia, nor are there standards for the practice of pharmaceutical care services. Lacking these core standards and frameworks limits the identification of learning gaps and development needs.

A recent study utilized the FIP GbCF v1 to assess its relevance to current local practice in Saudi Arabia (Alfaifi et al., 2022). The study showed that 84% of the competencies and behaviors included in the GbCF v1 are relevant to pharmacy practice in Saudi Arabia. However, some behaviors were found to require modification to be appropriate for the local needs of Saudi pharmacy practice. The work done by Alfaifi et al. helped clarify current practices and identified relevant competencies to include in a country-specific competency framework. However, data on ‘not relevant’ competencies are also important in order to identify gaps in practice. Previous studies have highlighted that, although some competencies in the GbCF were perceived as ‘not relevant’ to practice, stakeholders included them (or a modification of them) in their national competency framework to foster the development and improvement of pharmacy practice (Al-Haqan et al., 2021).

With the recent update and revision to the FIP GbCF and the launch of FIP DGs, this study aimed to adopt the GbCF v2 to evaluate the competencies’ relevance to current practice and to map ‘not relevant’ competencies to FIP DGs in order to recommend strategies for workforce and practice development and improvement.

## 2 Methods

The methodology for adopting the GbCF followed the methodologies of previous studies with the same aim as the current study (Udoh et al., 2018, Mucalo et al., 2016, Al-Haqan et al., 2021). This was conducted using four phases: Phase 1— translation, Phase 2— consensus panel validation, and Phase 3— national survey. Phase 4 was carried out after survey analysis and involved mapping ‘not relevant’ competencies (e.g. ‘Areas for improvement’) to the FIP DGs to identify priorities for development and improvement training.

### 2.1 Phase 1 – Translation

Phase 1 was carried out during April 2021. A back translation method was used by translating the English version of GbCF V2 into the Arabic language, the official language of the country. The translation was performed by a bilingual pharmacist with expertise in policy formation and pharmacy regulation, therefore, familiar with the terminology of the framework. The Arabic version of the framework was then back-translated by two bilingual translators, and the two versions were compared for accuracy and discrepancies. The final version of the Arabic framework was discussed and approved by the translators and the study authors who are also bilingual English-Arabic speakers.

### 2.2 Phase 2 – Validation

Phase 2 was undertaken in May 2021. Five online meetings were held with stakeholders representing various pharmacy sectors aiming to validate the Phase 1 outcome, i.e. accuracy of framework translation, as well as confirmation of initial consensus competencies and behavioral indicators relevant to Saudi practice. A purposive sampling technique was used to recruit pharmacists who were fluent as English/Arabic speakers with work experience in different pharmacy fields. The FIP GbCF -V2 in its Arabic translation was presented to these pharmacists to receive their comments about the clarity and accuracy of the framework translation, as well as whether the listed behaviors were relevant to the national context and inclusive regarding daily practice. Participants were requested to assess the relevance of each behavioral statement using a 4-point Likert scale, ranging from 1 = not relevant to 4 = highly relevant. The final consensus was reached when the participants of each group reached agreement on the degree of relevance of each behavioral indicator. Online meetings lasted 20–35 min on average and were facilitated by the study's principal investigator. Discussions were not audio recorded, but notes were taken by a scribe and the facilitator.

### 2.3 Phase 3 – National survey

Phase 3 was carried out between June and September 2021. This intended to assess the relevance of the FIP GbCF v2 behavioral statements at a national level across all settings. A cross-sectional online survey using Qualtrics Survey Software QSS (Qualtrics, Provo, UT, USA) was implemented. The original FIP GbCF v2 included 123 behavioral statements, while this survey excluded 61 statements because they were previously rated as “relevant “or “highly relevant” by participants in Phase 2. Hence, they were excluded to reduce the time to complete the questionnaire.

The questionnaire contained 71 questions. Eight questions related to participants’ demographics and the remaining 63 questions pertained to behavioral statements. A 4-point Likert scale (ranging between 1 = not relevant and 4 = highly relevant) was used to assess the relevance of each behavioral statement. Before distributing the questionnaire, it was piloted with a convenience sample of 10 pharmacists in various settings.

All pharmacists registered by SCFHS (n = 29,090) were eligible to take part in the national survey. The web-based questionnaire was sent as an anonymous link to representative authorities, including the Saudi Pharmaceutical Society (SPS) and Saudi Society of Clinical Pharmacy (SSCP). The link to the online questionnaire was emailed to members of both societies and posted on existing official Saudi pharmacy societies accounts on social media, such as Twitter. Pharmacists were also requested to forward the link to their social networks of eligible participants. Reminders were sent every couple of weeks for four months.

### 2.4 Data analysis

Data from Phase 2 validation were entered into an Excel spreadsheet and analyzed using frequency and percentages. Data were collected and reported in aggregates.

Data from the national survey were analyzed using IBM SPSS Statistics for Windows, version 27. Replies to the questionnaire were automatically coded and exported to SPSS using a survey software (Qualtrics, Provo, UT, USA). Demographic characteristics were summarized using frequencies and percentages. Respondents’ perceptions of relevance to behavioral statements were also summarized using frequencies and percentages. The four-point Likert scale was aggregated into two categories; the ‘highly relevant’ and ‘relevant’ ratings were condensed into one category designated as 'relevant,' while the ‘low relevance’ and ‘not relevant’ ratings were condensed into a second category named ‘not relevant’. The decision as to whether to identify a ‘not relevant’ competency as current practice was guided by published healthcare research (Udoh et al., 2018, Al-Haqan et al., 2021). Consensus on relevance to practice was attained when <25% of responses were in the ‘low relevant/not relevant’ ranks.

This analysis was performed in three steps. Step 1 noted overall relevance to practice where all responses (across all statements) were aggregated to evaluate if respondents identified any area for improvement. Step 2 highlighted whether relevance per cluster required a closer look as to which aspect of current practice was perceived as ‘not relevant’. Clusters with high ‘not relevant’ responses were further analyzed, and inferential analysis was conducted using the Pearson’s Chi-Square (χ^2^) test to evaluate the relationship between survey responses and respondents’ characteristics, such as the area of practice and length of practice. Areas for improvement were analyzed according to the following predictor variables: (a) years in practice: <3 years and >3 years; (b): practice setting: hospital, community, medical store, Saudi Food and Drug Administration, regulatory affairs, pharmaceutical companies, academia, and others. The χ^2^ tests were assessed using a statistical significance predefined as p < 0.05. Step 3 determined the relevance per competency group in order to identify perceived areas needing improvement.

### 2.5 Phase 4: FIP DGs priorities

Competency groups with ‘not relevant’ responses higher than the defined consensus level of ≥25% were mapped to the FIP DGs. FIP DGs were used as an analytical framework to map areas for improvement and identify priorities for improving current practice and workforce development. The mapping process was carried out by one author (AA) and another independent researcher who is familiar with the FIP DGs. Each worked independently and discrepancies were discussed until consensus was reached.

## 3 Results

### 3.1 Phase 1

The translated framework was incorporated into the survey statements. The survey was designed and distributed to include each statement in English and Arabic languages (Arabic version of the survey is available from the authors upon request).

### 3.2 Phase 2

This phase aimed to establish initial consensus on the relevance of the competencies and behaviors included in the FIP GbCFv2 to pharmacy practice in Saudi Arabia, as well as to verify the Arabic translation of the original English document. A total of 14 pharmacists with expertise in hospital pharmacy (n = 3), community pharmacy (n = 3), regulatory affairs (n = 1), Food and Drug Authority (n = 1), academia (n = 2), medical store (n = 2), and pharmaceutical companies (n = 2) agreed to participate. Participants found the translated statements equivalent to the original English statements. They all agreed that the translation was correct, and the Arabic words and phrases used were understandable.

Of the 124 statements, 63 statements were reported as ‘not relevant’ to current practice (no consensus, ≤25% of participants). Statements that were reported as ‘not relevant’ were considered to be representing areas for improvement, as well as gaps in providing pharmaceutical care to patients. Those statements were included in Phase 3, the national survey, to further investigate their relevancy from a wider pharmacy perspective.

### 3.3 Phase 3

A total of 224 participants accessed the survey; however, only 163 responses were valid, giving a response rate of 72.8%. Mean (±SD) age of respondents was 32.9 (±12.6). More than half of respondents (n = 110, 67.5%) were males, 62.0% (n = 101) were Saudi citizens, half (n = 82, 50.4%) had a post-graduate degree, 76.7%.8 (n = 125) had more than three years in practice, and half had a patient-facing role, either in a hospital (n = 45, 27%.6) or community pharmacy (n = 47, 28.8%). Respondents’ demographics and other characteristics are shown in Table 1 Participants’ responses to each behavioral statement are presented in Table 2.

**Table 1:** Demographics and other characteristics (N = 163).

Age mean (±SD)	32.9 (±12.6)
**Gender**	**N (%)**
Male	110 (67.5)
Female	53 (32.5)
**Nationality**	**N (%)**
Saudi	101 (62.0)
Non-Saudi	62 (38.0)
**Year of graduation**	**N (%)**
1970–1979	1(0.6)
1980–1989	2 (1.2)
1990–1999	15 (9.2)
2000–2009	46 (28.2)
2010–2021	97 (59.5)
**Country of undergraduate degree**	**N (%)**
Saudi	100 (61.3)
Others	63 (38.7)
**Post-graduate qualifications**	**N (%)**
Yes	82 (50.3)
No	81 (49.7)
**Years in practice mean (±SD)**	**7.9 (13.8)**
**Years in practice**	
1–3 year	38 (23.3)
>3 years	125 (76.7)
Practice site	N (%)
Hospital	45 (27.6)
Community pharmacy	47 (28.8)
Medical store	1 (0.6)
Saudi Food and Drug Administration	3 (1.8)
Regulatory affairs	4 (2.5)
Pharmaceutical companies	24 (14.7)
Academia	34 (20.9)
Other	5 (3.1)

**Table 2:** Participants’ responses to behavioural statements.

		Not relevant	Relevant	Total
**Cluster1: Pharmaceutical Public Health**				
Emergency response	2-Assist the multidisciplinary healthcare teams in emergency situations	47 (30.7)	106 (69.3)	153
Medicines information and advice	7- Identify sources, retrieve, evaluate, organise, assess and provide relevant and appropriate medicines information according to the needs of patients and clients	21 (14.4)	125 (85.6)	146

**Cluster 2: Pharmaceutical Care**				
Assessment of medicines	10- Retrieve relevant patient information (including drug history, or immunisation status for example) and record of allergies to medicines and Adverse Drug Reactions (ADR) in medication record	32 (23.2)	106 (76.8)	138
	11- Identify, prioritise, resolve and follow up on medicine-medicine interactions; medicine-disease interactions; medicine-patient interactions; medicines-food interactions	33 (24.3)	103 (75.7)	136
	12- Appropriately select medicines (e.g. according to the patient, hospital, government policy, etc)	21 (15.6)	114 (84.4)	135
Compounding medicines	13- Prepare pharmaceutical medicines (e.g. extemporaneous, cytotoxic medicines), determine the requirements for preparation (calculations, appropriate formulation, procedures, raw materials, equipment etc.)	54 (40.3)	80 (59.7)	134
	14- Compound under the good manufacturing practice for pharmaceutical (GMP) medicines	58 (43.3)	76 (56.7)	134
Dispensing	15- Accurately dispense medicines for prescribed and/or minor ailments, including an embedded checking process	37 (27.8)	96 (72.2)	133
	17- Dispense devices (e.g. Inhaler or a blood glucose meter)	45 (34.1)	87 (65.9)	132
	18- Appropriately validate prescriptions, ensuring that prescriptions are correctly interpreted and legal	30 (22.7)	102 (77.3	132
Medicines	25- Ensure appropriate medicines, route, time, dose, documentation, action, form and response for individual patients	35 (26.3)	98 (73.7)	133
	26- Package medicines to optimise safety (ensuring appropriate re-packaging and labelling of the medicines	38 (28.8)	94 (71.2)	132
Monitor medicines therapy	28- Apply therapeutic medicines monitoring and assess impact and outcomes (including objective and subjective measures)	46 (34.9)	86 (65.2)	132
Patient consultation and diagnosis	32- Assess and diagnose based on objective and subjective measures (where applicable)	56 (43.1)	74 (56.9)	130
	33- Evaluate, assess, and develop health literacy education and counselling on medicines and healthcare needs	33 (25.2)	98 (74.8)	131
	34- Discuss and agree with the patients the appropriate use of medicines, taking into account patent's preferences	36 (27.7)	94 (72.3)	130
	35- Document any intervention (e.g. document any allergies, medicines and food) in patient medicines history	31 (23.9)	99 (76.2)	130
	36- Obtain, reconcile, review, maintain and update relevant patient medication and disease history	38 (29.0)	93 (71.0)	131

**Cluster 3: Organisation and Management**				
Budget and reimbursement	38- Effectively set and apply budgets	45 (33.8)	88 (66.2)	133
	39- Manage appropriate claim for the reimbursement	56 (43.1)	74 (56.9)	130
	40- Ensure financial transparency	57 (44.2)	72 (55.8)	129
	41- Ensure proper reference sources for service reimbursement	53 (41.7)	74 (58.3)	127
Human resources management	42- Demonstrate organisational and management skills (e.g. plan, Organise and lead on medicines management; risk management, self-management, time management, people management, project management, policy management.)	28 (22.1)	99 (77.9)	127
	43- Identify and manage human resources and staffing issues	45 (34.9)	84 (65.1)	129
	45- Recognise the value of the pharmacy team and of a multidisciplinary team	25 (19.4)	104 (80.6)	129
Improvement of service	47- Identify, implement, and montior new services (according to local needs)	32 (24.8)	97 (75.2)	129
	48- Resolve, follow-up and prevent medicines related problems	29 (22.7)	99 (77.3)	128
Procurement	49- Access reliable information and ensure the most cost-effective medicines in the right quantities with the appropriate quality	29 (22.5)	100 (77.5)	129
	53- Identify and select reliable supplier(s)	39 (30.5)	89 (69.5)	128
	54- Select reliable supplies of high-quality products (including appropriate selection process, cost effectiveness, timely delivery)	40 (31.3)	88 (68.7)	128
	55- Supervise procurement activities	51 (40.5)	75 (59.5)	126
	56- Understand the trending methods and evaluation of tender bids	46 (35.9)	82 (64.1)	128
Supply chain management	57- Demonstrate knowledge in store medicines to minimise errors and maximise accuracy	30 (23.6)	97 (76.4)	127
	58- Verify the accuracy of rolling stocks	51 (40.5)	75 (59.5)	126
	59- Ensure effective stock management and running of service with the dispensary	43 (33.9)	84 (66.1)	127
	60- Ensure logistics of delivery and storage	39 (30.9)	87 (69.1)	126
	63- Mitigate risk of medicines shortages and stock outs through liaison and appropriate communication with healthcare staff, healthcare stakeholders, clients/customers and patients	33 (26.4)	92 (73.6)	125
Workplace management	69- Recognise and manage pharmacy resources (e.g. Financial, Infrastructure)	44 (35.8)	79 (64.2)	123

**Cluster 4: Professional/Personal**				
Communication skills	71- Communicate effectively with health and social care staff, support staff, patients, carer, family relatives and clients/customers, using lay terms and checking understanding	33 (25.9)	94 (74.1)	127
	72- Tailor communication that is appropriate to the patient’s needs (including health literacy, cultural or language barriers, social needs, and emotional status)	35 (27.8)	91 (72.2)	126
Continuing Professional Development (CPD)	75- Engage with students/interns/residents	25 (19.7)	102 (80.3)	127
	79- Identify if expertise is needed outside current scope of knowledge	35 (27.8)	91 (72.2)	126
Digital literacy	83- Identify, manage, organise, store, and share digital information	35 (27.8)	91 (72.2)	126
	84- Critically appraise, analyse, evaluate, and/or interpret digital information and their sources	41 (32.5)	85 (67.5)	126
Interprofessional collaboration	88- Participate, collaborate, advise in therapeutic decision-making, and use appropriate referral in a multi-disciplinary team	33 (26.2)	93 (73.8)	126
Leadership and self-regulation	92- Apply assertiveness skills (inspire confidence)	26 (20.6)	100 (79.4)	126
	96- Develop, implement and monitor innovative ideas	28 (22.2)	98 (77.8)	126
Legal and regulatory practice	101- Apply the principals of business economics and intellectual property rights, including the basics of patent interpretation	41 (32.8)	84 (67.2)	125
	102- Be aware of and identify the new medicines coming to the market	38 (30.4)	87 (69.6)	125
	106- Recognize the steps needed to bring a medical device or medicine a to the market, including the safety, quality, efficacy, and pharmacoeconomic assessments of the product	40 (32.3)	84 (67.7)	124
Professional and ethical practice	107- Demonstrate awareness and employment of local/national codes of ethics	16 (12.9)	108 (87.1)	124
	110- Comply with patient privacy legislation, including documentation of information	28 (22.6)	96 (77.4)	124
	111- Consider available evidence and support the patient to make informed choices about medicine use	27 (21.9)	96 (78.1)	123
	112- Obtain patient consent (it can be implicit in occasion)	37 (29.8)	87 (70.2)	124
	113- Recognise professional limitation of self and others in the team	30 (24.2)	94 (75.8)	124
	115- Demonstrate awareness of socially accountable practice (including cultural and social needs; cultural safety, respect, and responsiveness; diversity, equity and inclusiveness)	32 (25.8)	92 (74.2)	124
Quality assurance and research in the workplace	116- Apply research findings and understand the benefits/risk (e.g. pre-clinical, clinical trials, experimental clinical-pharmacological research and risk management)	32 (25.8)	92 (74.2)	124
	117- Audit quality of service (meet local and national standards and specifications)	30 (24.2)	94 (75.8)	124
	120- Ensure medicines are not counterfeit and adhere to quality standards	25 (20.7)	96 (79.3)	121
	121- Identify and evaluate evidence-based to improve the use of medicines and services	28 (22.8)	95 (77.2)	123
	122- Identify, investigate, conduct, supervise and Support research at the workplace (enquiry-driven practice)	31 (25.0)	93 (75.0)	124
	123- Implement, conduct and maintain a reporting system of pharmacovigilance (e.g. report Adverse Drug Reactions)	26 (20.9)	98 (79.1)	124
	124- Initiate and implement audit and research activities	31 (25.2)	92 (74.8)	123

### 3.4 Test of relevancy

#### 3.4.1 Overall relevance

**Fig. 2:**
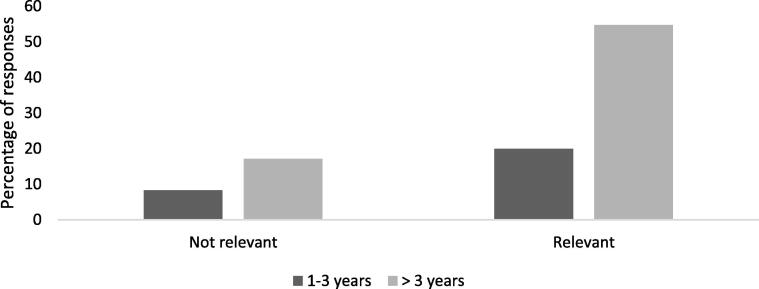
Percentages of responses per years in practice.

**Fig. 3:**
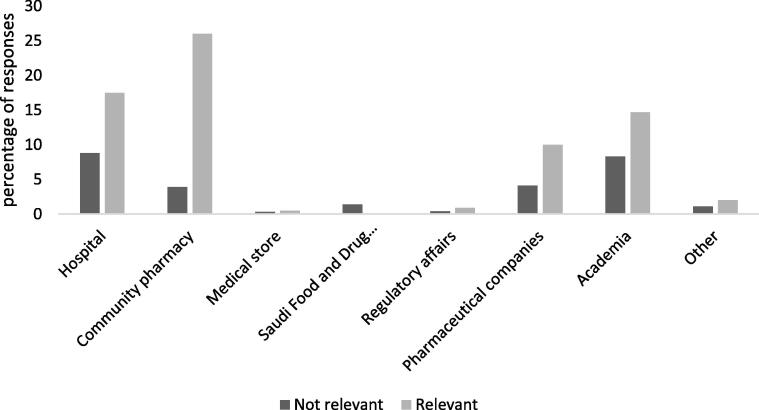
Percentages of responses per practice setting.

Responses in aggregate (across all statements) showed that 71.7% of all responses were in the ‘relevant’ category, while 28.3% of all responses were in the ‘not relevant’ category. This indicates areas for improvement in respondents’ performance or practice. It was found that respondents with more than three years in practice were more likely to perceive some of the behavioral statements to be ‘not relevant’ to their current practice (P < 0.001) (Fig. 2). On the other hand, respondents working in hospitals and academia were found to perceive some behavioral statements as ‘not relevant’ to their practice, while those practicing in community and other settings found them 'relevant' (P < 0.001) (Fig. 3).

#### 3.4.2 Relevance per cluster

Overall, clusters 1 (Pharmaceutical Public Health) and 4 (Professional/Personal) showed higher relevance to practice compared to clusters 2 and 3 (Table 3). Further analysis for the ‘not relevant’ responses showed that pharmacists who had more than three years in practice perceived clusters 1, 2, and 4 to be ‘not relevant’ to their current practice, compared to those who had less than three years of experience (p = 0.39, p < 0.001, and p < 0.001; respectively). On the other hand, pharmacists in academia perceived clusters 1 and 2 as ‘not relevant’ to their practice compared to other practice settings (p = 0.21, and p = 0.005; respectively). Moreover, pharmacists working in hospitals perceived clusters 3 and 4 less relevant to their practice than those in other practice settings (p < 0.001, and p = 0.37; respectively).

**Table 3:** Cluster relevancy.

Cluster	Not relevant	Relevant
	Responses N (%)	Responses N (%)
Cluster 1: Pharmaceutical Public Health	68 (22.8)	231 (77.2)
Cluster 2: Pharmaceutical Care	623 (29.4)	1500 (70.7)
Cluster 3: Organisation and Management	815 (31.9)	1739 (68)
Cluster 4: Professional/Personal	783 (25.1)	2333 (74.9)

#### 3.4.3 Relevance per competency

**Fig. 4:**
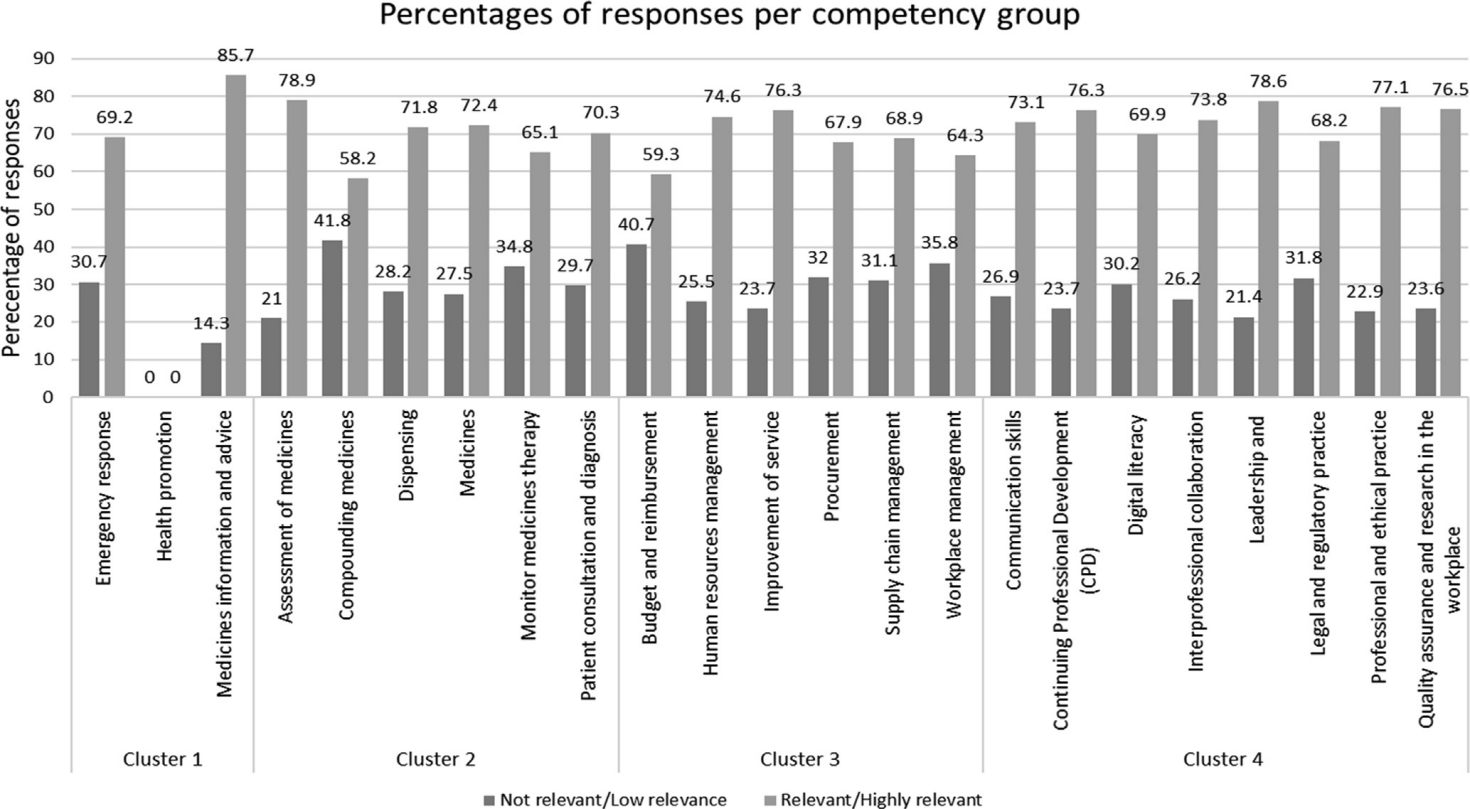
Percentages of responses per competency groups.

Fifteen out of the 23 competency groups had higher responses in the ‘not relevant’ category than the defined consensus level of ≥25% (Fig. 4). These competency groups are: emergency response, compounding medicines, dispensing medicines, monitoring medicine therapy, patient consultation and diagnosis, budget and reimbursement, human resources management, procurement, supply chain management, workplace management, communications, digital literacy, inter-professional collaboration, and legal and regulatory practice.

### 3.5 Phase 4

#### 3.5.1 FIP DGs priorities

The ‘not relevant’ competencies were mapped to FIP DGs to identify priority areas for improving current practice and workforce development (Table 4). Table 4 shows that the most frequently mapped DGs were DG5 (mapped to 7 competencies), and DG8 and DG11 (each mapped to 6 competencies). None of the ‘not relevant’ competencies were mapped to DGs 1, 2, 16 and 17.

**Table 4:** Mapping of ‘not relevant’ competencies to FIP DGs.

**Cluster**	**DG**																				
	DG1 Academic Capacity	DG2 Early career training strategy	DG 3 Quality assurance	DG4 Advanced and Spacilist development	DG5 Competency development	DG6 Leadership development	DG7 Advanced integrated services	DG8 Working with others	DG9 CPD strategies	DG10 Equity & Equality	DG11 Impact & outcomes	DG12 Pharmacy intelligence	DG13 Policy development	DG14 Medicines expertise	DG15 People-centred care	DG16 Communicable diseases	DG17 Antimicrobial stewardship	DG18 Access to medicines, devices, & services	DG19 Patient safety	DG20 Digital health	DG21 Sustainability in pharmacy
**Cluster1: Pharmaceutical Public Health**																					
Emergency response								**X**													

**Cluster 2: Pharmaceutical Care**																					
Compounding medicines			**X**		**X**									**X**							
Dispensing			**X**		**X**													**X**	**X**		
Medicines					**X**		**X**	**X**						**X**	**X**				**X**		
Monitor medicines therapy							**X**				**X**			**X**					**X**		
Patient consultation and diagnosis					**X**		**X**	**X**			**X**			**X**	**X**				**X**		

**Cluster 3: Organisation and Management**																					
Budget and reimbursement											**X**		**X**								**X**
Human resources management				**X**		**X**															
Procurement			**X**		**X**						**X**							**X**			**X**
Supply chain management			**X**					**X**			**X**							**X**			**X**
Workplace management																					**X**

**Cluster 4: Professional/Personal**																					
Communication skills					**X**			**X**							**X**						
Digital literacy					**X**						**X**									**X**	
Interprofessional collaboration								**X**													
Legal and regulatory practice					**X**								**X**	**X**				**X**			

Total	**0**	**0**	**4**	**1**	**8**	**1**	**3**	**6**	**0**	**0**	**6**	**0**	**2**	**5**	**3**	**0**	**0**	**4**	**4**	**1**	**4**

## 4 Discussion

Continued expansion of the pharmacist’s role necessitates continuous evaluation of current practice to identify strategies for improvement. Although a recent study used the FIP GbCF v1 in the Saudi context (Alfaifi et al., 2022), the competencies that were added/modified in GbCF v2 of the framework justified validating the new version in the current research (Ahmad et al., 2020). To our knowledge, this is the first study in the Eastern Mediterranean Region that uses both the FIP GbCF v2 and DGs to recommend strategies for workforce practice development and improvement.

The study involved four stages: translation, consensus panel validation, a national survey, and identification of FIP DGs priorities. Similar methodologies were employed in published literature from other countries including Croatia, Kuwait, Japan, Ireland and Indonesia (Mucalo et al., 2016, Al-Haqan et al., 2020, Meilianti et al., 2021, Al-Haqan et al., 2021, Arakawa et al., 2020, The Pharmaceutical Society of Ireland. CORE COMPETENCY FRAMEWORK for Pharmacists, 2013).

Our findings showed overall agreement on the relevance to practice of 71.7% of the behavioral statements included in this survey. Findings indicated higher levels of relevance to practice in pharmaceutical public health (cluster 1) and in personal/professional behaviors (cluster 4). This result aligned to study results in the previous work conducted in Saudi Arabia using GbCF v1 (Al-Arifi, 2013). This could be due to the fact that the majority of participants have been practicing in their respective sectors for more than 3 years. Hence, they were more likely to be involved in public health-related services and be focused on the professional/personal cluster competencies as they move up their career ladder. This contrasts with foundation-level pharmacists who are likely to perform pharmaceutical care-related tasks (cluster 2). A low relevance to organization and management (cluster 3) was previously reported in Japan, Kuwait and several African countries (Udoh et al., 2018, Al-Haqan et al., 2020, Arakawa et al., 2020). In the local context, the low relevance to organization and management was previously reported in terms of lacking leadership development strategies and programs at the undergraduate education and training levels (Almaghaslah and Alsayari, 2021). For example, in the community pharmacy sector, a lack of managerial skills stems from the limited availability of formal management programs (Al-Arifi, 2013). It’s been suggested that more effort should be directed towards developing administrative skills and leaderships skills, especially at the undergraduate education and early career stages, to enable pharmacists to take lead positions as they advance in their practice areas (Al-Arifi, 2013).

Regarding the level of engagement between sectors, the study results revealed that pharmacists in academia found the pharmaceutical public health (cluster 1) and pharmaceutical care (cluster 2) less relevant. A similar finding was previously reported in Japan (Alfaifi et al., 2022). This finding is likely a result of the nature of work at pharmacy colleges in Saudi Arabia. With the exception of pharmacy practice and clinical pharmacy departments, other pharmaceutical sciences departments are solely involved in teaching and research-based activities, rather than being involved in more patient-centered pharmaceutical care. On the other hand, pharmacists working in hospital sectors found the organization and management (cluster 3) and personal professional (cluster 4) clusters to be less relevant. This is likely because these pharmacists devote their time to more patient-centered pharmaceutical care.

The assessment of relevance per competency group identified 15 competency groups as not relevant, i.e., scored more than 25% in “not relevant” category. Two of these 15 competencies (emergency response and digital health) were newly added to the GbCF v2 and not included in GbCF v1, therefore not previously evaluated. Although emergency response was among the “not relevant” competency group, the recent covid-19 pandemic has highlighted the role of pharmacists in the management of the outbreak in Saudi Arabia (Al-Arifi, 2013). Moreover, the use of digital health resources helped pharmacists contribute to many initiatives, including health education and promotion, medication delivery/dispensing, medication reconciliation, patient counselling, emergency preparedness, and training for self-management in times of health crisis. Hence, the incorporation of these competencies will allow pharmacists to play a crucial role in emergency response as frontline healthcare providers (Ahmad et al., 2020).

Other competencies that were perceived as “not relevant” were compounding medicines, dispensing medicines, monitoring medicine therapy, patient consultation and diagnosis. This was consistent with previous research in the region. For example, patient consultation and diagnosis were also reported to be irrelevant in Saudi Arabia and Kuwait, as these are not common practice and not regulated nationally (Al-Haqan et al., 2021, Alfaifi et al., 2022).

The following competencies— budget and reimbursement, human resources management, procurement, supply chain management and workplace management— were also found ‘not relevant’ to current practice, similar to the previous Saudi study. The explanation is that these competencies are usually conducted at organizational and institutional levels, rather than at a departmental level (Alfaifi et al., 2022). Hence, most of the staff pharmacists in the different pharmacy sectors found them irrelevant to their practice.

The present study showed that competencies related to communications were perceived as ‘not relevant.’ This was inconsistent with findings from previous studies and will require more research to understand respondents’ perceptions toward these statements. Moreover, similar to the present study, inter-professional collaboration was previously identified as a pharmacy workforce development need in Saudi Arabia. Collaborative teamwork among healthcare professionals is a top priority for fulfilling the Saudi healthcare Vision 2030 for achieving better health outcomes (Almaghaslah et al., 2021). This finding draws attention to the importance of inter-professional collaboration during early education and training levels. Finally, legal and regulatory practices were also perceived as ‘not relevant’ to practice. Although a previous study in Saudi Arabia disagreed with statements in this competency, this result was found consistent with the results from Kuwait (Al-Haqan et al., 2021, Alfaifi et al., 2022). More research is needed to investigate the relevance of legal and regulatory competency to current practice in Saudi Arabia.

Identification of ‘not relevant’ competencies highlighted some gaps in current practice. These gaps may guide strategic planning to improve pharmacists’ performance and to expand services provided to patients, as well as to strengthen the healthcare system. Linking these gaps to global development goals may help to align local improvement strategies with the global vision for the advancement of the pharmacy profession worldwide. Therefore, to determine which strategies need to be developed to improve current practice, the ‘not relevant’ competencies were mapped to FIP DGs.

The present study showed that DG5: Competency Development was at the top of the priority list. This goal was identified as a priority goal in Kuwait and Lebanon as well (International Pharmaceutical Federation, 2022). The lack of a competency framework for pharmacists was also identified in a previous study as a priority for workforce development in Saudi Arabia (Almaghaslah and Alsayari, 2021). A competency framework for pharmacists could contribute to advancing both undergraduate and postgraduate pharmacy education in terms of curriculum development and learning outcomes. It supports the structuring of a continuous professional development framework and assists pharmacists to attain better practice and therapeutic outcomes (Almaghaslah and Alsayari, 2021). Therefore, future development projects may focus on developing competency frameworks (foundational and advanced).

Other goals that were identified as priority areas were DG 8: Working with Others and 11: Impact and Outcome. DG8 was also identified as a practice priority in Egypt (International Pharmaceutical Federation, 2022). The added value of a pharmacist in multidisciplinary intra- and inter-professional healthcare teams was highlighted in several studies (Bond and Raehl, 2007, Leary et al., 2019, Zhai et al., 2016). However, findings from the present study were similar to other studies in the region that showed the limited participation of pharmacists in multidisciplinary healthcare services (Chamoun et al., 2020). Therefore, there is a need to develop and implement structured and evidence-based strategies, systems, and protocols for integrated professional services and to advocate for pharmacists as active members in these services.

Additionally, in the present study, DG 11 was frequently mapped to competencies related to organization and management. The lack of pharmacy management competencies were identified in this study as well as in previous research (Alfaifi et al., 2022). This may hinder the impact of pharmacists within health systems. A positive impact and outcome for pharmaceutical services starts with good organization and management policies. It is recommended to implement systems to measure and monitor service impacts and outcomes that are based on agreed definitions and standards, along with quality and performance indicators (International Pharmaceutical Federation, 2022).

Although some DGs were less frequently mapped to the identified ‘not relevant’ competencies, the mapping process shed light on the top priorities for pharmacy practice and workforce development needs. This may guide stakeholders in developing future improvement projects. It also may guide future research to provide a better understanding on establishing a system of evaluation and an audit of pharmaceutical services.

### 4.1 Limitations

This investigation has several limitations. The low sample size might affect the generalizability of the findings, bearing in mind that this response rate is similar to previous studies using the same competency framework. The length of the survey with its 63 behavioral statements resulted in incomplete responses that were excluded from the final analysis. Underrepresentation of certain sectors, including regulatory affairs and pharmaceutical companies, might have resulted in an inaccurate representation of the relevancy of the framework to pharmacists working in these areas of practice. Self- administration of the survey might also have affected the validity of the responses. The present study utilized the FIP GbCF v2, which is intended to support early career pharmacists and foundation level competencies, and this may not reflect a comprehensive overview of the current practice. Therefore, a future researcher may aim to investigate the relevance of the FIP advanced competency framework (GADF) to identify development goals related to advanced practice in the local context.

## 5 Conclusion

The current study assessed the relevance of the FIP GbCF v2 in pharmacy practice in Saudi Arabia. It highlighted the gaps in competency groups and the development goals that need attention. This work could be used as starting point towards developing strategies, systems, and protocols to advance pharmacy practice in Saudi Arabia.

## Declaration of Competing Interest

The authors declare that they have no known competing financial interests or personal relationships that could have appeared to influence the work reported in this paper.

## References

[b0005] Ahmad A., Alkharfy K.M., Alrabiah Z., Alhossan A. (2020). Saudi Arabia, pharmacists and COVID-19 pandemic. J. Pharm. Policy Pract..

[b0010] Al-Arifi M.N. (2013). The Managerial Role of Pharmacist at Community Pharmacy Setting in Saudi Arabia. Pharmacol. Pharm..

[b0015] Alfaifi S., Arakawa N., Bridges S. (2022). The relevance of the International Pharmaceutical Federation Global Competency Framework in developing a country-level competency framework for pharmacists: A cross-sectional study. Explor. Res. Clin. Soc. Pharm..

[b0020] Al-Haqan A., Smith F., Al-Taweel D., Bader L., Bates I. (2020). Using a global systematic framework tool to guide the advancement of the pharmacy workforce education and training on a national level. Res. Soc. Adm. Pharm..

[b0025] Al-Haqan A., Smith F., Bader L., Bates I. (2021). Competency development for pharmacy: Adopting and adapting the Global Competency Framework. Res. Soc. Adm. Pharm..

[b0030] Almaghaslah D., Alsayari A., Almanasef M., Asiri A. (2021). A Cross-Sectional Study on Pharmacy Students’ Career Choices in the Light of Saudi Vision 2030: Will Community Pharmacy Continue to Be the Most Promising, but Least Preferred, Sector?. Int. J. Environ. Res. Public Health.

[b0035] Almaghaslah D., Alsayari A. (2021). Using a Global Systematic Framework Tool to Identify Pharmacy Workforce Development Needs: A National Case Study on Saudi Arabia. Risk Manag. Healthc. Policy.

[b0040] Almaghaslah D., Alsayari A., Asiri R., Albugami N. (2019). Pharmacy workforce in Saudi Arabia: Challenges and opportunities: A cross-sectional study. Int. J. Health Plann. Manage..

[b0045] AlRuthia Y., Alsenaidy M.A., Alrabiah H.K., AlMuhaisen A., Alshehri M. (2018). The status of licensed pharmacy workforce in Saudi Arabia: A 2030 economic vision perspective. Hum. Resour. Health.

[b0050] Andrew B., Ben G., Andreia B., Gabriella C. (2012). Validated Competency Framework for Delivery of Pharmacy Services in Pacific-Island Contries. J. Pharm. Pract. Res..

[b0055] Arakawa N., Yamamura S., Duggan C., Bates I. (2020). The development of a foundation-level pharmacy competency framework: An analysis of country-level applicability of the Global Competency Framework. Res. Soc. Adm. Pharm..

[b0060] Bond C.A., Raehl C.L. (2007). Clinical pharmacy services, pharmacy staffing, and hospital mortality rates. Pharmacother. J. Hum. Pharmacol. Drug Ther..

[b0065] Chamoun N., Usta U., Karaoui L.R., Salameh P., Hallit S., Shuhaiber P., Henaine A.-M., Akiki Y., Zeenny R.M., Iskandar K. (2020). Current trends in hospital pharmacy practice in Lebanon. Hosp. Pharm..

[b0070] FIP, 2020. FIP Global Competency Framework. https://www.fip.org/file/4805.

[b0075] HRSD, 2020. Renationalisation of the pharmacy profession. Published 2020. https://hrsd.gov.sa/ar/news/ His Excellency the Minister of Labor and Social Development issues a decision to nationalize the pharmacy profession.

[b0080] International Pharmaceutical Federation, 2022. The FIP Development Goals Report 2021: Setting Goals for the Decade Ahead [Online]. Accessed January 24, 2022. https://www.fip.org/file/5095.

[b0085] International Pharmaceutical Federation, 2020. The FIP Development Goals: Transforming Global Pharmacy [Online]. Accessed August 14, 2021. https://www.fip.org/file/4793.

[b0090] Koehler T.C., Bok H., Westerman M., Jaarsma D. (2018). Research in Social and Administrative Pharmacy Developing a competency framework for pharmacy technicians: Perspectives from the field. Res. Soc. Adm. Pharm..

[b0095] Leary M.-H., Morbitzer K., Walston B.J., Clark S., Kaplan J., Waldron K., Valgus J., Falato C., Amerine L. (2019). Evaluation of targeted pharmacist interventions to reduce length of stay in an acute care practice model. Ann. Pharmacother..

[b0100] Meilianti S., Smith F., Ernawati D.K., Pratita R.N., Bates I. (2021). Pharmacy A country-level national needs assessment of the Indonesian pharmacy workforce. Res. Soc. Adm. Pharm..

[b0105] Meilianti S., Smith F., Bader L., Himawan R., Bates I. (2021). Competency-based education: Developing an advanced competency framework for Indonesian pharmacists. Front. Med..

[b0110] Meštrović A., Staničić Ž., Hadžiabdić M.O., Mucalo I., Bates I., Duggan C., Carter S., Bruno A., Košiček M. (2012). Individualized education and competency development of Croatian community pharmacists using the general level framework. Am. J. Pharm. Educ..

[b0115] Mucalo I., Hadžiabdić M.O., Govorčinović T., Šarić M., Bruno A., Bates I. (2016). The Development of the Croatian Competency Framework for Pharmacists. Am. J. Pharm. Educ..

[b0120] SFDA, 2012. Saudi Code of Pharmaceutical Promotional Practices in the Kingdome of Saudi Arabia.

[b0125] Shah S., Mclaughlin J.E., Eckel S.F., Mangun J., Hawes E. (2016). Evaluating the Quality of Competency Assessment in Pharmacy: A Framework for Workplace Learning. Pharmacy.

[b0130] Suwannaprom P., Kessomboon N. (2020). Development of pharmacy competency framework for the changing demands of Thailand ’ s pharmaceutical and health services. Pharm. Pract..

[b0135] The Pharmaceutical Society of Ireland, 2013. Core Competency Framework for Pharmacists 2013.

[b0140] Udoh A., Bruno A., Bates I. (2018). A survey of pharmacists’ perception of foundation level competencies in African countries. Hum. Resour. Health.

[b0145] Udoh A., Bruno-Tomé A., Ernawati D.K., Galbraith K., Bates I. (2021). The effectiveness and impact on performance of pharmacy-related competency development frameworks: A systematic review and meta-analysis. Res. Soc. Adm. Pharm..

[b0150] Zhai X., Gu Z., Liu X. (2016). Effectiveness of the clinical pharmacist in reducing mortality in hospitalized cardiac patients: a propensity score-matched analysis. Ther. Clin. Risk Manag..

